# Molecular Hydrogen as a Lewis Base in Hydrogen Bonds and Other Interactions

**DOI:** 10.3390/molecules25143294

**Published:** 2020-07-20

**Authors:** Sławomir J. Grabowski

**Affiliations:** 1 Kimika Fakultatea, Euskal Herriko Unibertsitatea UPV/EHU, and Donostia International Physics Center (DIPC), P.K. 1072, 20080 San Sebastian, Spain; s.grabowski@ikerbasque.org; Tel.: +34-943-018-187; 2 IKERBASQUE, Basque Foundation for Science, 48011 Bilbao, Spain

**Keywords:** hydrogen bond, σ-hole bond, π-hole bond, molecular hydrogen, dihydrogen stretching mode

## Abstract

The second-order Møller–Plesset perturbation theory calculations with the aug-cc-pVTZ basis set were performed for complexes of molecular hydrogen. These complexes are connected by various types of interactions, the hydrogen bonds and halogen bonds are most often represented in the sample of species analysed; most interactions can be classified as σ-hole and π-hole bonds. Different theoretical approaches were applied to describe these interactions: Quantum Theory of ‘Atoms in Molecules’, Natural Bond Orbital method, or the decomposition of the energy of interaction. The energetic, geometrical, and topological parameters are analysed and spectroscopic properties are discussed. The stretching frequency of the H-H bond of molecular hydrogen involved in intermolecular interactions is considered as a parameter expressing the strength of interaction.

## 1. Introduction

Three types of hydrogen bonds occur that differ in the character of the proton acceptor according to one of classifications [1,2]. The three centre–four electron (3c-4e) hydrogen bonds, marked as A-H···B, are characterised by a single atom centre proton donor (A), and also one centre occurs for a proton acceptor (B) [3,4]. The O-H···O, N-H···O, C-H···N, and numerous other hydrogen bonds are commonly known as 3c-4e systems. The latter designation informs of three centres in the system: A, H, and B, and of four electrons attributed to them: a σ-bond (A-H) and a lone electron pair of the proton acceptor centre (B). This is an approximate picture since various arrangements that are often designated as A-H···B hydrogen bonds possess a more complicated electron structure.

For two other types of hydrogen bonds, the multicentre proton acceptor may be specified. For one of these types, the π-electron systems play such a role [5]; acetylene or ethylene molecule and their derivatives as well as aromatic systems are examples of species that act as Lewis base units. The O-H···π and C-H···π interactions are classified as such hydrogen bonds. One can mention specific systems like the T-shaped structure of acetylene [6] or the hydrogen fluoride that acts as the proton donor being directed to the benzene ring [7]. Particularly, the C-H···π systems were a subject of numerous studies and discussions; they often occur in crystal structures [8]. The hydrogen bonds with σ-electrons acting as the proton acceptor are the other type of interactions [2,9,10]. For example, the molecular hydrogen that is approximately perpendicular to the A-H proton donating bond acts as the proton acceptor through its σ-electrons. However, the C-C σ-bonds may also play a role of multicentre Lewis base sites, in cycloalkanes, for example [11,12,13].

One can mention the hydrogen-bonded systems, where both the proton donor and the proton acceptor are multicentre systems [14], but usually the above-mentioned three types of hydrogen bonds are specified; A-H···B, A-H···π, and A-H···σ. It is worth to mention that such categorization may be performed for other interactions, not only for hydrogen bonds. For example, there are pnicogen bonds with the single centre and multicentre electron donors, π-electron, or σ-electron systems [15]. The same is observed for triel bonds where the triel atom, as boron or aluminum, for example, may interact as the Lewis acid centre with the single centre or multicentre electron donors [16]. It seems that such categorizations into three types of Lewis base centres may be performed for the majority of interactions, particularly for the σ-hole and π-hole bonds [15]. The pnicogen and triel bonds mentioned above are specific examples of these two latter interactions, respectively.

It seems that, among interactions mentioned above here, those with the molecular hydrogen playing a role of the Lewis base through its σ-electrons are very interesting and important ones. Various types of interactions of the molecular hydrogen are analysed in a context of the hydrogen storage materials [17,18,19]. The dihydrogen cleavage is another subject related to the hydrogen storage [20,21,22]. The H_2_ activation plays an important role in the latter process. There are numerous extended studies concerning such activation that proceeds mainly at transition metal centres. The frustrated Lewis pairs may also play the crucial role in the dihydrogen activation; the corresponding experimental [23,24] and theoretical studies [25,26] were carried out recently. One can refer to theoretical studies concerning the activation of molecular hydrogen at the single non-metal centres, as on the carbon centre in singlet carbenes [27] and on other non-metals as sulphur, selenium, phosphorus, and arsenic [28]. Referring to transition metals, the analysis of the H_2_ activation at coinage metals in fluorides, and the dihydrogen splitting was discussed recently, since a theoretical analysis for corresponding systems was performed [20].

Another group of studies related to the dihydrogen activation concerns its interactions with the lighter elements; these interactions are often classified as the above mentioned σ-hole and π-hole bonds [29,30,31,32,33,34,35]. For example, the A-H···σ interactions with the σ-electrons of dihydrogen were predicted, characterised theoretically and classified as hydrogen bonds for the first time several years ago [9]. The NH_4_^+^···H_2_ complex and the NH_4_^+^···(H_2_)_n_ clusters (*n* up to 8) as well as structures containing ammonia cation analogue where nitrogen is replaced by another element of the group 15 are systems where the existence of A-H···σ hydrogen bonds was detected [9]; similarly, such hydrogen bonds occur in the T-shaped H_2_···HF and H_2_OH^+^···H_2_ structures [10]. This is important that such interactions occur in structures analysed experimentally, for example, the T-shaped H_2_···HF [36,37,38,39] or the H_2_···HCO^+^ complex [40] were analysed, but they were not considered as the hydrogen bonded systems.

The halogen, pnicogen, chalcogen, and triel bonds are other examples of interactions in complexes of dihydrogen [15,41,42]; the latter species plays a role of the Lewis base unit in such complexes. The aim of this study was to analyse the broad spectrum of interactions in complexes of dihydrogen, most of them classified as the σ-hole and π-hole bonds. The reasons of such a choice of complexes to be analysed concern their importance in numerous processes, as, for example, the dihydrogen activation and cleavage mentioned earlier here; other reasons are discussed later here. The interactions analysed in this study are characterised by numerous geometrical, energetic, and topological parameters; the additional attention is paid to spectroscopic features.

## 2. Results and Discussion

### 2.1. The Strength of Interactions in Complexes of Dihydrogen

**Figure 1:**
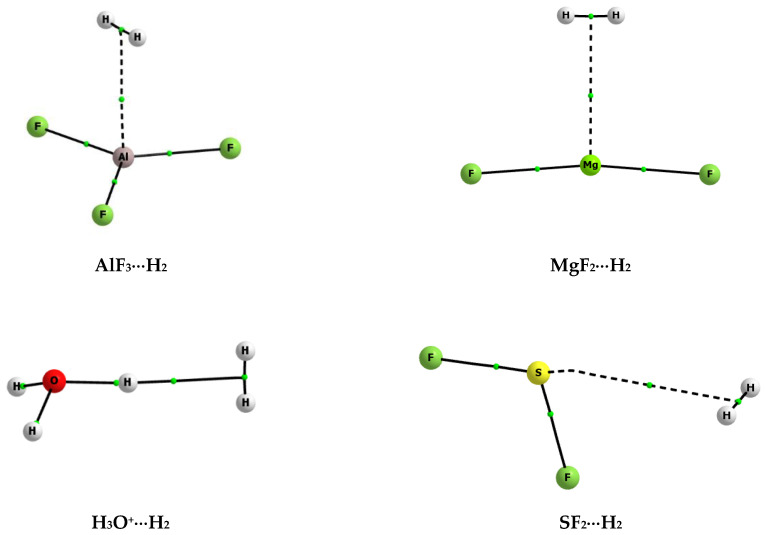
The molecular graphs of selected complexes analysed here; big circles correspond to attractors, small green circles to bond critical points, and continuous and broken lines to bond paths.

Figure 1 shows few examples of molecular graphs of complexes analysed here. The complexes linked by the triel, magnesium, hydrogen, and chalcogen bonds are presented where the aluminum, magnesium, hydrogen, and sulphur atoms act as the Lewis acid centres, respectively, since they are in contact with the σ-electrons of dihydrogen. These graphs as well as corresponding interactions are discussed later here.

Table 1 presents the binding and interaction energies, E_bin_ and E_int_, respectively [43]; the deformation energy, E_def_ [44], is also included as well as the basis set superposition error (BSSE) correction [45], for all complexes analysed in this study. Table 1 also presents a kind of interaction attributed to each complex that is considered here. Thus, one can see that a broad spectrum of interactions for complexes of molecular hydrogen is taken into account in this study. The following interactions are considered; triel [16,42], beryllium [46,47,48], magnesium [49,50,51,52,53], lithium [54,55,56], hydrogen [57,58], tetrel [59,60,61,62], pnicogen [35,41,63,64,65,66,67], chalcogen [68,69,70,71,72,73], and halogen [30,31,32,33,74,75] bonds. Some of them may be classified as the σ-hole bonds [29,30,31,32,33] that are links between the σ-hole and the electron rich region. The σ-hole is a region of the electron charge depletion at the atomic centre in the direction of the bond to this centre. If such depletion is sufficient, then the region of σ-hole is characterised by the positive electrostatic potential, EP, and the Lewis acid properties. Such regions occur for Si, P, S, and Cl centres in dihydrogen complexes discussed here, the corresponding tetrel, pnicogen, chalcogen, and halogen bonds are classified as the σ-hole bonds. It was discussed by Politzer and co-workers that regions at atomic centres in planar molecules or planar molecular fragments may occur which are also characterised by the positive EP; such regions are named as π-holes and corresponding interactions with Lewis bases as the π-hole bonds [32,33]. The triel bonds discussed in this study (Table 1) are classified as the π-hole bonds where regions of the positive EP occur at the boron or aluminum centre. The Lewis acid properties of the triel centres related to their positive EP regions were discussed in earlier studies [76,77] and it was found that, in a case of some of boron and aluminum trihalides, BX_3_ and AlX_3_, respectively, the regions of positive EP occur at halogen centres (σ-holes), in elongations of B/Al-X bonds. This is observed for the heavier Cl and Br halogen centres, but not for the fluorine atoms. This is in line with other studies where it was pointed out that fluorine acts as the Lewis acid only in very rare cases [30,75]. The other interactions discussed here, the hydrogen bond and the lithium bond, were also analysed and considered if they may be classified as the σ-hole bonds [56,78].

Such broad spectrum of Lewis acid centres interacting with dihydrogen was chosen, since in principle only for the transition metals their interactions with σ-electrons were analysed in detail. For other types of Lewis acid centres, such analyses are rather rare. Especially the non-metallic centres of the 14th, 15th, 16th, and 17th groups were not discussed earlier extensively as interacting with dihydrogen; one may mention only few studies [27,28]. The analysis of the broad spectrum of centres which interact with molecular hydrogen may be very important in studies concerning activation of the latter species and of its cleavage, in particular.

The interaction energy, E_int_, presented in Table 1 is defined as the difference between the energy of the complex and the sum of energies of interacting units; the geometries of units are those taken from the geometry of the complex. The binding energy, E_bin_, is defined in the same way as the difference between the energy of the complex and the sum of energies of interacting units. However the units of the complex are optimised separately. Hence, their geometries correspond to isolated species possessing configurations of energetic minima. Thus, one can see that E_bin_ takes into account the deformation of interacting units that results from complexation, in other words, E_bin_ − E_int_ = E_def_ [44].

It was found, for the hydrogen bonded complexes, that E_def_, which is characterised by the positive value, increases with the increase of the strength of interaction. The same is observed here, the greatest E_def_ value exceeding 7 kcal/mol occurs for the BH_3_···H_2_ complex linked by the triel bond. For the tetrel, pnicogen, chalcogen, and halogen bonds considered here, the deformation energy is lower than 0.005 kcal/mol, there is only one exception, for the PFH_2_···H_2_ complex it amounts 0.01 kcal/mol (Table 1). Such negligible deformation energies for the mentioned above interactions are accompanied by weak intermolecular interactions, the −E_int_ (interaction energy corrected for BSSE) values below 1 kcal/mol are observed here. It is worth to mention that at least slightly stronger interactions should occur for heavier halogen, pnicogen, chalcogen, and tetrel centres, since it was found in numerous studies that the strength of interaction for the same group elements often increases with the increase of the atomic number [31,32,33,59,78,79,80,81].

The observation that mainly metal centres are linked with molecular hydrogen [21,22] are partly confirmed here. For the results presented in Table 1, the strongest interactions are those defined as triel, beryllium, and magnesium bonds. For the BH_3_···H_2_ complex, the −E_int_ value amounts to 12.6 kcal/mol and −E_bin_ is equal to 5.4 kcal/mol; that indicates large deformations resulting from complexation, which was mentioned above here. The strong Lewis acid properties of the boron centre were discussed in earlier studies, particularly for the BH_3_···H_2_ complex [42], it was also later explained that the strong interaction of dihydrogen with the boron hydride is mainly related to the σ_H2_ → n(2p-B) orbital–orbital overlap [82]. The lower Lewis acid properties of boron are observed for BF_3_ species that is explained by back bonding effect [83] and by other specific characteristics of this compound [77]. The weaker total interactions in the AlH_3_···H_2_ and AlF_3_···H_2_ complexes than in the BH_3_···H_2_ complex may be explained by the stronger polarization of the Al-H and Al-F bonds than of the B-H bond [76,77] that results in stronger electrostatic and weaker charge transfer interactions in the aluminum complexes. The hydrogen bonds may be classified as rather weak or at most as medium in strength interactions; the latter ones occur in complexes of the ammonia and hydronium cations acting as the Lewis acid units, −E_int_ values exceed 2 kcal/mol and 5 kcal/mol, respectively, here. The BSSE correction (Table 1) does not exceed 1 kcal/mol, it is between 0.19 kcal/mol and 0.71 kcal/mol for triel bonded complexes. For the remaining complexes, this value is lower than 0.3 kcal/mol; there is only one exception: for the SiF_4_···H_2_ complex, the BSSE correction is equal to 0.38 kcal/mol.

### 2.2. The H_2_ Stretch Mode in Complexes of Dihydrogen

Table 2 presents other characteristics of complexes analysed here. For example, the H-H bond lengths are collected. One can see that the complexation leads to elongations of these bonds, since the H-H bond length for the isolated molecular hydrogen amounts 0.737 Å. The greatest elongation, up to 0.799 Å, is observed for the BH_3_···H_2_ complex where the strongest interaction, i.e., triel bond, occurs. For remaining complexes, such elongation is not significant. For weak interactions discussed in the previous section, the interaction energy does not exceed 1 kcal/mol; for tetrel, pnicogen, chalcogen, and halogen bonds, the H-H bond length does not exceed 0.74 Å; in other words, the elongation is equal to or smaller than 0.002 Å. The relationship between the strength of interaction expressed by E_int_ and the H-H bond length is observed. However, it is only rough dependence. For the second order polynomial regression, the squared correlation coefficient, R^2^ = 0.946, if the BH_3_···H_2_ complex mentioned above is excluded from this regression as being far from other values of the sample considered; however, if this complex is not excluded, then R^2^ = 0.975.

**Figure 2:**
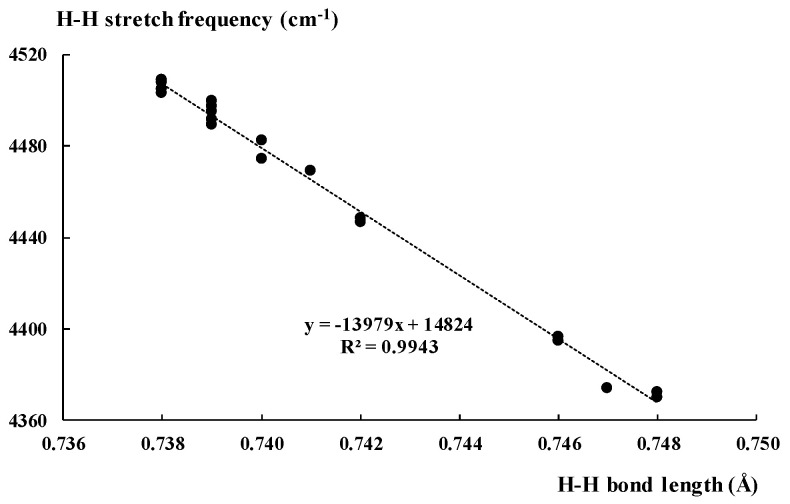
The linear correlation between the H-H bond length (Å) and the corresponding H-H bond stretching frequency (cm^−1^).

The spectroscopic parameters of dihydrogen seem to be characterised by the greater sensitivity to the environment effects, i.e., to interactions with the Lewis acid units, than the geometrical parameters. The calculated H_2_ stretching frequency for isolated dihydrogen amounts to 4517.6 cm^−1^ (Table 2). The experimental fundamental ground tone vibration of H_2_, determined to an accuracy of 2 × 10^−4^ cm^−1^ from Doppler-free laser spectroscopy in the collision less environment of a molecular beam, is equal to 4161.16632(18) cm^−1^ [84]. The theoretically determined vibrations collected in Table 2 indicate the shift to the lower frequencies, red-shift, in relation to the free dihydrogen, that corresponds to the elongation of the H-H bond. Figure 2 presents the linear correlation between the stretching vibration and the H-H bond length for the complexes analysed here. The BH_3_···H_2_ complex is excluded, since it is characterised by values being far from those of other complexes. The rejection of one point from this correlation is statistically justified and even required since ν_HH_ for the BH_3_···H_2_ complex is equal to 3630.8 cm^−1^; that is far away from the range for remaining complexes (4370.2 cm^−1^–4508.9 cm^−1^). However, if this point is included, thus the linear relationship is even better, R^2^ = 0.9995.

Table 2 also presents the intensities corresponding to the vibrations collected. Approximately the greater intensity is observed for the stronger interaction, accompanied by the greater elongation of the H-H bond and the greater red-shift of the corresponding stretching mode. However, there is no any clear dependence between these parameters. This is because of the various kinds of interactions in the complexes considered here. Anyway, if only strong interactions: triel, beryllium, and magnesium bonds are considered (without the BH_3_···H_2_ complex, because of the same statistical reasons as before), thus the exponential relationship between the H-H stretching frequency and the corresponding intensity occurs (R^2^ = 0.970).

**Figure 3:**
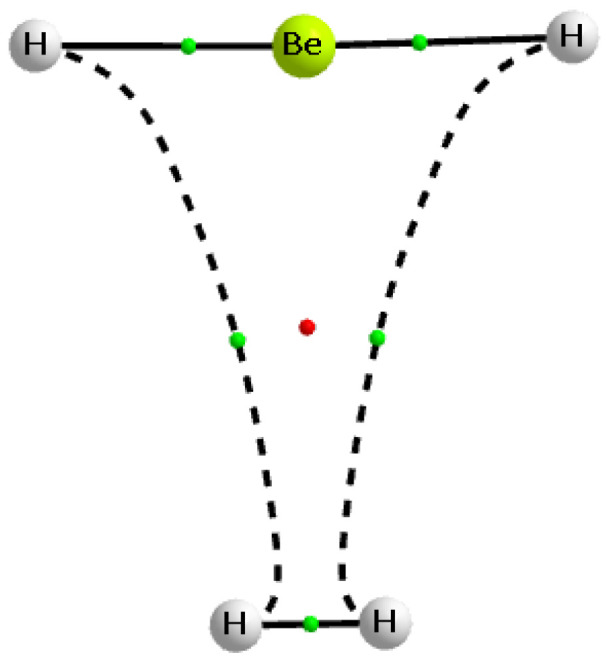
The molecular graph of the BeH_2_···H_2_ complex; big circles correspond to attractors, small green circles to bond critical points, small red circle to the ring critical point, and continuous and broken lines to bond paths.

Table 2 also presents the charges of molecular hydrogen in complexes analysed. Almost in all cases, it is positive indicating the outflow of the electron charge from dihydrogen that results from complexation. Approximately the greater outflow occurs for stronger interactions; the complex of BH_3_···H_2_ is an example where huge outflow of this charge is observed, leading to the charge of +0.217 au for dihydrogen in this complex. In two complexes, the electron charge shifts from the Lewis acid unit to the Lewis base unit outweigh the reverse processes. This leads to the negative charge of the molecular hydrogen in the complex. In the case of the SiFH_3_···H_2_ complex, this charge amounts to −0.005 au only; that may be connected with the weakness of the corresponding interaction and not sufficient accuracy of calculations. However, the case of the BeH_2_···H_2_ complex is much more expressive, the negative charge of dihydrogen amounts here −0.269 au. It may be explained by the significant outflow of the negative charge from H-atoms of the beryllium hydride to the dihydrogen. Figure 3 shows the molecular graph of this complex; one can see two H···H bond paths linking the beryllium hydride with the dihydrogen. In the isolated BeH_2_ and H_2_ species, the neutral H-atoms occur for the dihydrogen, while for the beryllium hydride H-atoms are negatively charged. The electron charge shifts lead to the negatively charged H-atoms of dihydrogen and to the positive charges of all atoms of the Lewis acid unit, BeH_2_. Thereby these H···H links may be treated as dihydrogen bonds [2,85,86,87], since they occur between H-atoms of the opposite charges.

Table 2 shows energies of the most important orbital–orbital interactions for each of complexes considered here. Low energies of this type are observed for interactions mentioned above here as weak ones, for halogen, chalcogen, pnicogen, and tetrel bonds. These are energies below 0.7 kcal/mol corresponding to the σ_H2_ → σ_AZ_^*^ overlaps. A designates here an atom connected with the Z Lewis acid centre (halogen, chalcogen, pnicogen, or tetrel). Stronger orbital–orbital interactions are observed for some of the complexes linked by the hydrogen bond. These are the σ_H2_ → σ_AH_^*^ overlaps, as it was described in earlier study [2]. For the NH_4_^+^···H_2_ and H_3_O^+^···H_2_ complexes, the energies coresponding to the σ_H2_ → σ_NH_^*^ and σ_H2_ → σ_OH_^*^ overlaps are equal to 4.0 kcal/mol and 16.9 kcal/mol, respectively. The greatest energies occur for the triel, beryllium, and magnesium bonds, they correspond to the σ_H2_ → n(Z)^*^overlaps; n designates here the lone electron pair orbital, while Z is the B, Al, Be, or Mg centre. The huge energy of 282.1 kcal/mol is observed for the strongest interaction in the BH_3_···H_2_ complex.

### 2.3. The A-H Stretching Mode in Hydrogen Bonded Complexes

Table 3 presents characteristics of complexes linked by the A-H···B hydrogen bond. There are five complexes linked by this kind of interaction in the sample considered here. The A-H proton donating bond spectroscopic characteristics are presented in this table, with the A-H stretching frequency and the intensity of corresponding mode. These values for the isolated Lewis acid units and the corresponding values in complexes are presented. One can see that all hydrogen bonds analysed in this study are classified as the red-shifted hydrogen bonds [57,58], since in all cases the complexation leads to the elongation of the proton-donating bond and consequently to the shift of the A-H stretching frequency to lower values (the red-shift) and to the increase of the corresponding mode intensity. The greatest changes are observed for the strongest interactions in the NH_4_^+^···H_2_ and H_3_O^+^···H_2_ complexes.

The percentage increase of the length of A-H bond that results from complexation is also presented in Table 3. It is calculated according to the equation given below.

(1) $$AH_{inc}\%=[\frac{\left(r_{AH}-r_{AH}^{0}\right)}{r_{AH}^{0}}]\;100\%$$

In this equation, $r_{AH}$ and $r_{AH}^{0}$ designate the lengths of the proton donating bond, in the complex and in the isolated Lewis acid unit not involved in any interaction, respectively. It is worth to mention that the bond length increase presented above has been introduced in an earlier study as a measure of the hydrogen bond strength, together with another measure—so-called complex parameter based on the AH bond lengths and on the topological parameters [88].

**Figure 4:**
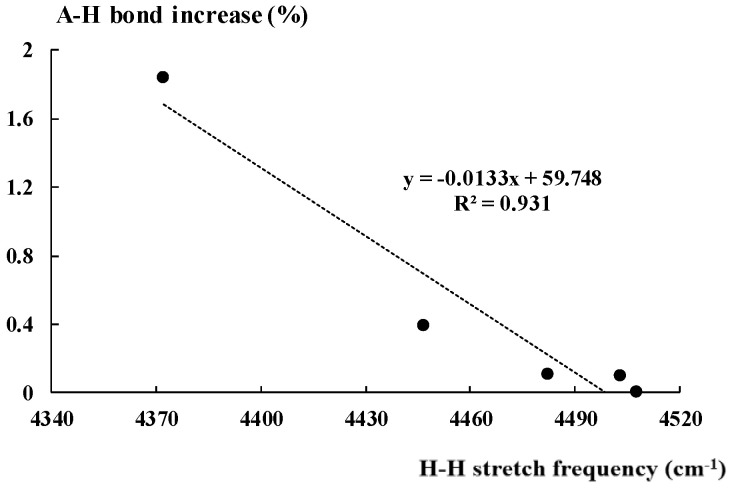
The linear correlation between the H-H bond stretching frequency (cm^−1^) and the corresponding percentage increase of the A-H bond length.

For the complex of hydronium cation, this increase is equal to 1.8%, for the NH_4_^+^···H_2_ complex it amounts to 0.4%, while for other three cases considered, it is much lower. There are relationships between parameters describing the dihydrogen unit and those corresponding to the Lewis acid. Figure 4 presents the linear correlation between the stretching frequency of the H-H bond of molecular hydrogen in the hydrogen bonded complex and the percentage increase of the A-H proton donating bond (Equation (1)) discussed above. In spite of various kinds of hydrogen bonds analysed here, the squared correlation coefficient R^2^ = 0.93. However, this correlation should be treated with caution, since only five complexes in this sample are considered; thus, it is not sufficient from the statistical point of view.

It is worth to mention that the similar parameter to this one presented above (Equation (1)) which is based on stretching frequencies may be introduced (Equation (2)).

(2) $$\nu_{AH}\%=[\frac{\left(\nu_{AH}^{0}-\nu_{AH}\right)}{\nu_{AH}^{0}}]\;100\%$$

Similarly to designations concerning the bond lengths of Equation (1), in Equation (2), $\nu_{AH}$ and $\nu_{AH}^{0}$ designate the A-H stretching frequencies of the proton donating bond, in the complex and in the isolated Lewis acid unit, respectively. This parameter (Equation (2)) may be also treated as a measure of the hydrogen bond strength, since it correlates well with bond lengths´ parameter (Equation (1)), the squared linear correlation coefficient, R^2^ is equal to 0.988.

### 2.4. The Topological Parameters

The Quantum Theory of ‘Atoms in Molecule’ (QTAIM) approach [89,90] was applied to analyse intermolecular interactions. Figure 1 and Figure 3 present molecular graphs of the selected complexes discussed here. For the majority of complexes, the Lewis acid and Lewis base units are linked by the bond path that connects the bond critical point (BCP) of dihydrogen with the Lewis acid centre. The BCP assigned to this bond path may be useful to characterise the corresponding interaction, since, in numerous studies, various correlations between QTAIM, energetic, and geometrical parameters were found [90,91,92,93,94]. In few cases of complexes analysed here, another picture occurs, since there is no simple bond path that links dihydrogen BCP with the single Lewis base centre; the BeH_2_···H_2_ complex is an example discussed earlier here (Figure 3).

Table 4 presents selected QTAIM parameters for few complexes analysed here; only those linked by the relatively strong interactions (−E_int_ > 2 kcal/mol) are presented, since for the remaining complexes the low values of the electron density at BCP, *ρ*_BCP_, are observed. These low values are accompanied by the positive Laplacian of the electron density at the BCP, ▽^2^*ρ*_BCP_, and positive H_BCP_ values. In other words, such QTAIM parameters characterise weak interactions between closed shell systems [89,90]. For the results collected in Table 4, all ▽^2^*ρ*_BCP_ values are positive, but in two cases H_BCP_ is negative, for the BH_3_···H_2_ and H_3_O^+^···H_2_ complexes. This means that the corresponding interactions may be treated as at least partly covalent in nature. In the case of the former complex the strongest interaction is observed (Table 1) in the sample of species analysed here, while for the latter complex linked by the hydrogen bond, E_int_ = −5.5 kcal/mol. This value is comparable with the interaction that occurs for the dimer of water linked by the O-H···O hydrogen bond [58].

**Figure 5:**
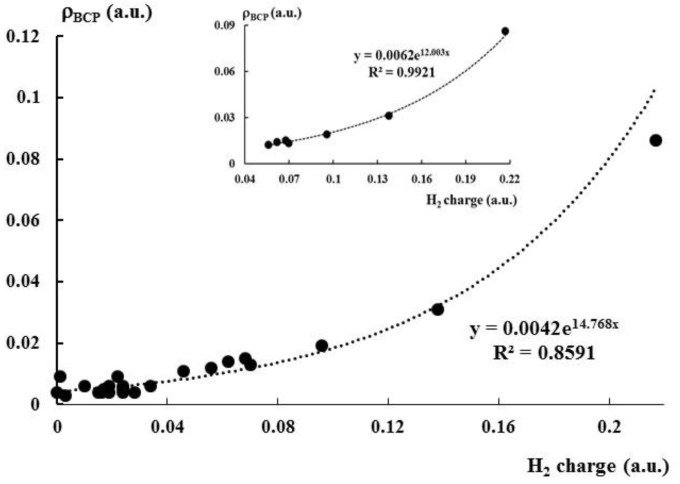
The exponential relationship between the H_2_ charge (au) and the electron density at the intermolecular BCP (au); the greater scatter plot concerns the whole sample, while the smaller one concerns the sub-set of strong interactions.

This is worth to mention that clear dependencies between QTAIM parameters and other characteristics are observed only for stronger interactions, i.e., for those collected in Table 4. Figure 5 presents exponential relationship between the positive charge of dihydrogen in the complex and the electron density at the BCP corresponding to the intermolecular bond path. It was found in numerous studies that *ρ*_BCP_ expresses the strength of interaction [89,90,91,92,93,94]. Hence, the exponential relationship of Figure 5 shows that the greater outflow of the electron charge from dihydrogen occurs for stronger interactions. Much better dependence is observed if only strong interactions collected in Table 4 are considered (small scatter plot at the top of Figure 5), for the whole sample where the complexation leads to the outflow of the electron charge from the dihydrogen the correlation is worse (the greater scatter plot of Figure 5), partly because of the lower accuracy of results for weaker interactions.

### 2.5. The Decomposition of the Energy of Interaction

The decomposition of the energy of interaction [95,96] was also performed here, but similarly as the QTAIM analysis, only for stronger interactions, since, for weaker ones, the terms of energy resulting from decomposition are not determined with sufficient accuracy. Table 5 presents the results of decomposition. One can see that the electrostatic and orbital, ΔE_elstat_ and ΔE_orb_, respectively, are two most important attractive interactions. The dispersion interaction energy, ΔE_disp_, is much lower for all complexes considered, except for the Li^+^···H_2_ complex where ΔE_disp_ is comparable with the ΔE_elstat_ term, even the former one outweighs slightly the latter one (this concerns absolute values). One can also see that for complexes analysed that are characterised by stronger interactions, the orbital interaction is much more important (possesses ¨more negative¨ value) than the electrostatic interaction; only for the MgF_2_···H_2_ complex, the ΔE_elstat_ and ΔE_orb_ terms are comparable. The importance of the orbital interaction shows that the stabilisation of complexes of dihydrogen that are linked by stronger interactions is connected mainly with the electron charge shifts.

## 3. Conclusions

The complexes of molecular hydrogen are analysed here, where various types of interactions occur; triel, magnesium, beryllium, lithium, hydrogen, tetrel, chalcogen, pnicogen, and halogen bonds link molecular hydrogen with different Lewis acid units considered in this study. The triel, beryllium, magnesium, and lithium bonds are stronger than the remaining interactions. The charge assisted hydrogen bonds in complexes of ammonia, and hydronium cations are exceptions, since they are relatively strong, the hydrogen bond in the complex of hydronium cation possesses partly covalent character.

Different parameters are discussed here, among them, the stretching frequency of the H-H bond which may be considered as a measure of the strength of interaction. It correlates with other parameters describing the strength of interaction. It was found that for the hydrogen-bonded systems, the characteristics of the A-H proton donating bond correlate with those of the molecular hydrogen.

Almost for all dihydrogen complexes described, the bond path links the BCP of H_2_ unit with the Lewis acid centre; such a path may be treated as the characteristic feature of such interactions. The results discussed here show that the activation of dihydrogen that can lead to its cleavage is more pronounced for stronger interactions.

## 4. Computational Details

The calculations for complexes analysed in this study have been performed with the use of Gaussian16 set of codes [97]. The second-order Møller–Plesset perturbation theory (MP2) [98] with the aug-cc-pVTZ basis set [99] were applied. Frequency calculations have been carried out at the same MP2/aug-cc-pVTZ level and it was found that the optimised structures of complexes correspond to the energetic minima, similarly as the Lewis acid and Lewis base units optimised separately that constitute the complexes considered.

The Counterpoise Correction mentioned before here is the most often applied approach to estimate the basis set superposition error, BSSE [45,100]. It is defined by Equation (3) given below.

ΔE_CP_ (BSSE) = E(A)^A^ + E(B)^B^ – E(A)^A^^∪B^ – E(B)^A^^∪B^ (3)

The designations A and B in parentheses correspond to monomers that constitute the complex analysed, and superscripts designate the basis sets applied in calculations. The latter means that for A and B monomers the corresponding A and B basis sets may be used as well as that the complex A∪B basis set may be applied. The ΔE_CP_ value (BSSE correction) is positive, since the energy calculated at complex basis set (A∪B) is lower than the energy calculated with the use of the monomer basis set (A or B). The geometries of A and B monomers are those in the complex in the energetic minimum.

The Gaussian16 program was also used to calculate the electron charge shifts resulting from complexation within the CHelpG approach [101]. The CHelpG charges are fitted to the electrostatic molecular potential (EMP) with the use of the grid-based method. It was justified that the application of the CHelpG method based on well-defined EMP values produces much better estimates of the intermolecular charge transfer than other population analyses [102].

The Quantum Theory of ‘Atoms in Molecules’ (QTAIM) [89,90] was applied to analyse characteristics of bond critical points (BCPs) of the intermolecular contacts; in a case of the A-H···σ hydrogen bonds, these are the H···σ contacts. The AIMAll program [103] was used to perform corresponding QTAIM calculations. The most important orbital–orbital interactions in complexes analysed were evaluated within the Natural Bond Orbital, NBO, approach. The Gaussian NBO Version 3.1 incorporated in the Gaussian16 set of codes [97] was used to calculate energies of these interactions within the HF/aug-cc-pVTZ level of approximation.

The BP86-D3/TZ2P level was applied to perform decomposition energy calculations. It means that the BP86 functional [104,105] with the Grimme dispersion corrections [106] and the uncontracted Slater-type orbitals (STOs) as basis functions with triple-ζ quality for all elements [107] were applied. The decomposition energy calculations [95,96] were carried out with the use of the ADF2013.01 program [108] for geometries of complexes optimized at the MP2/aug-cc-pVTZ level. The total interaction energy for the energy partitioning applied here is composed of terms according to the Equation (4), given below.

ΔE_int_ = ΔE_elstat_ + ΔE_Pauli_ + ΔE_orb_ + ΔE_disp_ (4)

The term ΔE_elstat_ is usually attractive (negative) and it corresponds to the quasi-classical electrostatic interaction between the unperturbed charge distributions of atoms. The Pauli repulsion, ΔE_Pauli_, is the energy change associated with the transformation from the superposition of the unperturbed electron densities of the isolated fragments to the wave function that properly obeys the Pauli principle through antisymmetrisation and renormalization of the product wave function. This energy term (positive) that corresponds to the repulsion interaction comprises the destabilizing interactions between electrons of the same spin on either fragment. The orbital interaction energy, ΔE_orb_, corresponds to the charge transfer and polarization effects, briefly speaking to the electron charge shifts resulting from complexation. The dispersion interaction energy, ΔE_disp_, is also included in this decomposition scheme.

## Figures and Tables

**Table 1:** The energetic characteristics of complexes analysed (in kcal/mol), the interaction and binding energy corrected for basis set superposition error (BSSE) (E_int_ and E_bin_, respectively), the deformation energy (E_def_), and the BSSE; the type of interaction is also indicated (Bond). The results of MP2/aug-c-pVTZ calculations, i.e., second-order Møller–Plesset perturbation theory (MP2) with the aug-cc-pVTZ basis set are presented, the same level calculations are shown in other tables.

Lewis Acid	Bond	E_int_	E_bin_	E_def_	BSSE
AlF_3_	Triel	−4.78	−4.02	0.76	0.71
AlH_3_	Triel	−2.80	−2.58	0.22	0.19
BF_3_	Triel	−0.67	−0.66	0.01	0.39
BH_3_	Triel	−12.62	−5.39	7.23	0.53
BeF_2_	Beryllium	−1.17	−0.96	0.21	0.28
BeH_2_	Beryllium	−0.48	−0.46	0.02	0.06
MgF_2_	Magnesium	−3.57	−3.28	0.29	0.30
MgH_2_	Magnesium	−1.15	−1.07	0.08	0.07
Li^+^	Lithium	−5.84	−5.81	0.03	0.04
HCCH	Hydrogen	−0.30	−0.30	0.00	0.12
HF	Hydrogen	−0.94	−0.94	0.00	0.18
HCN	Hydrogen	−0.45	−0.45	0.00	0.17
NH_4_^+^	Hydrogen	−2.44	−2.42	0.02	0.15
H_3_O^+^	Hydrogen	−5.53	−5.29	0.24	0.25
SiF_4_	Tetrel	−0.41	−0.41	0.00	0.38
SiFH_3_	Tetrel	−0.57	−0.57	0.00	0.22
PFH_2_	Pnicogen	−0.92	−0.91	0.01	0.30
P(CN)H_2_	Pnicogen	−0.54	−0.54	0.00	0.16
S(CN)_2_	Chalcogen	−0.87	−0.87	0.00	0.19
SF_2_	Chalcogen	−0.60	−0.60	0.00	0.21
Cl_2_	Halogen	−0.52	−0.52	0.00	0.15
HCCCl	Halogen	−0.37	−0.37	0.00	0.1
(NC)CCCl	Halogen	−0.46	−0.46	0.00	0.13
NCCl	Halogen	−0.46	−0.46	0.00	0.11
CF_3_Cl	Halogen	−0.32	−0.32	0.00	0.12

**Table 2:** The characteristics of complexes analysed, r(H_2_)—the H-H bond length (Å), ν_HH_—H-H bond stretching frequency (in cm^−1^), I_HH_—intensity of the H-H stretching mode (km mol^−1^), q(H_2_)—the charge of dihydrogen in the complex (in au), and E_NBO_—the energy of the most important orbital–orbital interaction in the complex considered (in kcal/mol). *

Lewis Acid	r(H_2_)	ν_HH_	I_HH_	q(H_2_)	E_NBO_
AlF_3_	0.748	4370.2	34.42	0.096	20.0
AlH_3_	0.747	4374.2	21.19	0.068	21.5
BF_3_	0.739	4497.46	1.95	0.01	1.1
BH_3_	0.799	3630.8	19.69	0.217	282.1
BeF_2_	0.741	4469.32	4.13	0.022	3.8
BeH_2_	0.739	4491.49	2.17	−0.269	1.4
MgF_2_	0.746	4396.43	11.52	0.056	11.1
MgH_2_	0.742	4448.55	6.2	0.024	4.5
Li^+^	0.746	4394.79	43.41	0.07	4.8
HCCH	0.738	4508	1.06	0	0.4
HF	0.74	4482.4	4.11	0.046	1.3
HCN	0.738	4503	2.8	0.017	0.7
NH_4_^+^	0.742	4446.8	32.61	0.062	4
H_3_O^+^	0.748	4372.3	45.12	0.138	16.9
SiF_4_	0.738	4505.2	0.45	0.003	0.1
SiFH_3_	0.739	4497.3	1.16	−0.005	0.7
PFH_2_	0.74	4474.5	1.07	0.001	0.2
P(CN)H_2_	0.739	4497.6	1.5	0.015	0.5
S(CN)_2_	0.739	4489.2	1.8	0.024	0.4
SF_2_	0.739	4495.2	1.83	0.019	0.5
Cl_2_	0.739	4499.6	2.47	0.034	0.5
HCCCl	0.738	4507.9	0.71	0.016	0.2
(NC)CCCl	0.738	4505	2.15	0.019	0.2
NCCl	0.738	4505	1.78	0.028	0.2
CF_3_Cl	0.738	4508.9	0.69	0.024	0.2

* characteristics of the isolated dihydrogen calculated at the same level as complexes collected; H_2_ stretching frequency ν_HH_^0^ = 4517.6 cm^−1^, H-H bond length r_0_(H_2_) = 0.737 Å.

**Table 3:** The spectroscopic characteristics of the proton-donating bond in hydrogen bonded systems; the A-H bond stretching frequency (cm^−1^)—ν_AH_, with the corresponding stretching mode intensity (km mol^−1^)—I_AH_, and the A-H bond length r_AH_ (in Å); these values for the AH bond not involved in H-bond interaction are given in parentheses, $AH_{inc}\%$—the percentage increase of the AH bond length that results from complexation (Equation (1)).

L. Acid	ν_AH_	I_AH_	r_AH_	AH_inc_%
HCCH	3430.7 (3433.7)	118.24 (95.57)	1.062 (1.062)	0
HF	4085.9 (4126.0)	252.44 (120.59)	0.923 (0.922)	0.11
HCN	3466.2 (3468.5)	117.44 (77.03)	1.065 (1.064)	0.09
NH_4_^+^	3476.2 (3527.2)	310.1 (193.57)	1.026 (1.022)	0.39
H_3_O^+^	3253.7 (3686.1)	1066.52 (481.4)	0.998 (0.980)	1.84

**Table 4:** The topological characteristics of the intermolecular bond critical point, BCP, (in au); *ρ*_BCP_—the electron density at BCP, ▽^2^*ρ*_BCP_—the Laplacian of this electron density, and H_BCP_—the corresponding total electron energy density at BCP.

Lewis Acid	*ρ*_BCP_	▽^2^*ρ*_BCP_	H_BCP_
AlF_3_	0.019	0.084	0.001
AlH_3_	0.015	0.050	0
BH_3_	0.086	0.073	−0.061
MgF_2_	0.012	0.070	0.003
Li^+^	0.013	0.072	0.004
NH_4_^+^	0.014	0.038	0
H_3_O^+^	0.031	0.048	−0.006

**Table 5:** The energy terms for selected complexes resulting from the decomposition of the energy of interaction (in kcal/mol); ΔE_Pauli_—the Pauli repulsion, ΔE_elstat_—the electrostatic term, ΔE_orb_—the orbital interaction energy, ΔE_disp_—the dispersion interaction energy term, and ΔE_int_—the total interaction energy.

Lewis Acid	ΔE_Pauli_	ΔE_elstat_	ΔE_orb_	ΔE_disp_	ΔE_int_
AlF_3_	15.04	−8.19	−10.98	−1.24	−5.37
AlH_3_	11.54	−6.25	−8.04	−0.72	−3.47
BH_3_	83.58	−33.84	−66.13	−1.35	−17.74
MgF_2_	7.66	−5.53	−5.11	−1.5	−4.48
Li^+^	3.39	−2.69	−6.31	−2.96	−8.57
NH_4_^+^	3.11	−1.7	−3.85	−0.94	−3.38
H_3_O^+^	8.01	−3.52	−11.21	−0.69	−7.41

## References

[1] Grabowski S.J. (2011). What is the Covalency of Hydrogen Bonding?. Chem. Rev..

[2] Grabowski S.J. (2013). Dihydrogen bond and X-H···σ interaction as sub-classes of hydrogen bond. J. Phys. Org. Chem..

[3] Weinhold F., Landis C. (2005). Valency and Bonding, A Natural Bond Orbital Donor—Acceptor Perspective.

[4] Weinhold F., Klein R.A. (2012). What is a hydrogen bond? Mutually consistent theoretical and experimental criteria for characterizing H-bonding interactions. Mol. Phys..

[5] Desiraju G.R., Steiner T. (1999). The Weak Hydrogen Bond in Structural Chemistry and Biology.

[6] Scheiner S., Grabowski S.J. (2002). Acetylene as a potential hydrogen-bond proton acceptor. J. Mol. Struct..

[7] Baiocchi F.A., Williams J.H., Klemperer W. (1983). Molecular beam studies of hexafluorobenzene, trifluorobenzene, and benzene complexes of hydrogen fluoride. The rotational spectrum of benzene-hydrogen fluoride. J. Phys. Chem..

[8] Nishio M., Hirota M., Umezawa Y. (1998). The CH/π Interaction: Evidence, Nature and Consequences.

[9] Szymczak J.J., Grabowski S.J., Roszak S., Leszczynski J. (2004). H···σ interactions—An ab initio and ‘atoms in molecules’ study. Chem. Phys. Lett..

[10] Grabowski S.J. (2007). Hydrogen Bonds with π and σ Electrons as the Multicenter Proton Acceptors: High Level Ab Initio Calculations. J. Phys. Chem. A.

[11] Rozas I., Alkorta I., Elguero J. (1997). Unusual hydrogen bonds: H···pi interactions. J. Phys. Chem. A.

[12] Galano A., Alvarez-Idaboy J.R., Vivier-Bunge A. (2007). Non-alkane behavior of cyclopropane and its derivatives: Characterization of unconventional hydrogen bond interactions. Theor. Chem. Acc..

[13] Grabowski S.J. (2019). A-H···σ Hydrogen Bonds: Dihydrogen and Cycloalkanes as Proton Acceptors. ChemPhysChem.

[14] Grabowski S.J., Sokalski W.A., Leszczynski J. (2004). Is a π···H^+^···π Complex Hydrogen Bonded?. J. Phys. Chem. A.

[15] Grabowski S.J. (2017). Hydrogen bonds, and σ-hole and π-hole bonds—Mechanisms protecting doublet and octet electron structures. Phys. Chem. Chem. Phys..

[16] Grabowski S.J. (2020). Triel bond and coordination of triel centres – Comparison with hydrogen bond interaction. Coord. Chem. Rev..

[17] Hamilton C.W., Baker R.T., Staubitz A., Manners I. (2009). B-N compounds for chemical hydrogen storage. Chem. Soc. Rev..

[18] Keaton R.J., Blacquiere J.M., Baker R.T. (2007). Base Metal Catalyzed Dehydrogenation of Ammonia−Borane for Chemical Hydrogen Storage. J. Am. Chem. Soc..

[19] Staubitz A., Besora M., Harvey J.N., Manners I. (2008). Computational Analysis of Amine− Borane Adducts as Potential Hydrogen Storage Materials with Reversible Hydrogen Uptake. Inorg. Chem..

[20] Grabowski S.J., Ruipérez F. (2016). Dihydrogen bond interactions as a result of H_2_ cleavage at Cu, Ag and Au centres. Phys. Chem. Chem. Phys..

[21] Kubas G.J. (2001). Metal Dihydrogen and σ-Bond Complexes—Structure, Theory, and Reactivity.

[22] Crabtree R.H. (2005). The Organometallic Chemistry of the Transition Metals.

[23] Stephan D.W., Erker G. (2014). Frustrated Lewis pair chemistry of carbon, nitrogen and sulfur oxides. Chem. Sci..

[24] Stephan D.W. (2009). Frustrated Lewis pairs: A new strategy to small molecule activation and hydrogenation catalysis. Dalton Trans..

[25] Rokob T.A., Bakó I., Stirling A., Hamza A., Pápai I. (2013). Reactivity models of hydrogen activation by frustrated Lewis pairs: Synergistic electron transfers or polarization by electric field?. J. Am. Chem. Soc..

[26] Pérez P., Yepes D., Jaque P., Chamorro E., Domingo L.R., Rojas R.S., Toro-Labbé A. (2015). A computational and conceptual DFT study on the mechanism of hydrogen activation by novel frustrated Lewis pairs. Phys. Chem. Chem. Phys..

[27] Frey G.D., Lavallo V., Donnadieu B., Schoeller W.W., Bertrand G. (2007). Facile splitting of hydrogen and ammonia by nucleophilic activation at a single carbon center. Science.

[28] Grabowski S.J. (2015). Cleavage of hydrogen by activation at a single non-metal centre towards new hydrogen storage materials. Phys. Chem. Chem. Phys..

[29] Clark T., Hennemann M., Murray J.S., Politzer P. (2007). Halogen bonding: The σ-hole. J. Mol. Model..

[30] Politzer P., Lane P., Concha M.C., Ma Y., Murray J.S. (2007). An overview of halogen bonding. J. Mol. Model..

[31] Politzer P., Riley K.E., Bulat F.A., Murray J.S. (2012). Perspectives on halogen bonding and other σ-hole interactions: Lex parsimoniae (Occam’s Razor). Comput. Theor. Chem..

[32] Politzer P., Murray J.S., Clark T. (2010). Halogen bonding: An electrostatically-driven highly directional noncovalent interaction. Phys. Chem. Chem. Phys..

[33] Politzer P., Murray J.S., Clark T. (2013). Halogen bonding and other σ-hole interactions: A perspective. Phys. Chem. Chem. Phys..

[34] Scheiner S. (2013). Detailed Comparison of the Pnicogen Bond with Chalcogen, Halogen, and Hydrogen Bonds. Int. J. Quantum Chem..

[35] Scheiner S. (2013). The Pnicogen Bond: Its Relation to Hydrogen, Halogen, and Other Noncovalent Bonds. Acc. Chem. Res..

[36] Jucks K.W., Miller R.E. (1987). Infrared Stark spectroscopy of the hydrogen–HF binary complex. J. Chem. Phys..

[37] Moore D.T., Miller R.E. (2003). Dynamics of hydrogen–HF complexes in helium nanodroplets. J. Chem. Phys..

[38] Moore D.T., Miller R.E. (2003). Solvation of HF by molecular hydrogen: Helium nanodroplet vibrational spectroscopy. J. Phys. Chem. A.

[39] Moore D.T., Miller R.E. (2004). Rotationally Resolved Infrared Laser Spectroscopy of (H_2_)*_n_*-HF and (D_2_)*_n_*-HF (*n* = 2−6) in Helium Nanodroplets. J. Phys. Chem. A.

[40] Bieske E.J., Nizkorodov S.A., Bennett F.R., Maier J.P. (1995). The infrared spectrum of the H_2_–HCO^+^ complex. J. Chem. Phys..

[41] Grabowski S.J., Alkorta I., Elguero J. (2013). Complexes between Dihydrogen and Amine, Phosphine, and Arsine Derivatives. Hydrogen Bond versus Pnictogen Interaction. J. Phys. Chem. A.

[42] Fau S., Frenking G. (1999). Theoretical investigation of the weakly bonded donor—Acceptor complexes X_3_B—H_2_, X_3_B—C_2_H_4_, and X_3_B—C_2_H_2_ (X = H, F, Cl). Mol. Phys..

[43] Piela L. (2007). Ideas of Quantum Chemistry.

[44] Grabowski S.J., Sokalski W.A. (2005). Different types of hydrogen bonds: Correlation analysis of interaction energy components. J. Phys. Org. Chem..

[45] Boys S.F., Bernardi F. (1970). The calculation of small molecular interactions by the differences of separate total energies. Some procedures with reduced errors. Mol. Phys..

[46] Yáñez M., Sanz P., Mó O., Alkorta I., Elguero J. (2009). Beryllium Bonds, Do They Exist?. J. Chem. Theory Comput..

[47] Eskandari K. (2012). Characteristics of beryllium bonds; a QTAIM study. J. Mol. Model..

[48] Mó O., Yáñez M., Alkorta I., Elguero J. (2012). Modulating the Strength of Hydrogen Bonds through Beryllium Bonds. J. Chem. Theory Comput..

[49] Yang X., Li Q., Cheng J., Li W. (2013). A new interaction mechanism of LiNH_2_ with MgH_2_: Magnesium bond. J. Mol. Model..

[50] McDowell S.A.C., Maynard S.J. (2016). A computational study of model hydrogen-, halogen-, beryllium-and magnesium-bonded complexes of aziridine derivatives. Mol. Phys..

[51] Li S.Y., Wu D., Li Y., Yu D., Liu J.Y., Li Z.R. (2016). Insight into structural and π–magnesium bonding characteristics of the X_2_Mg···Y (X = H, F.; Y = C_2_H_2_, C_2_H_4_ and C_6_H_6_) complexes. RSC Adv..

[52] Tama R., Mó O., Yáñez M., Montero-Campillo M.M. (2017). Characterizing magnesium bonds: Main features of a non-covalent interaction. Theor. Chem. Acc..

[53] Montero-Campillo M.M., Sanz P., Mó O., Yáñez M., Alkorta I., Elguero J. (2018). Alkaline-earth (Be, Mg and Ca) bonds at the origin of huge acidity enhancements. Phys. Chem. Chem. Phys..

[54] Kollman P.A., Liebman J.F., Allen L.C. (1970). The Lithium Bond. J. Am. Chem. Soc..

[55] McDowell S.A.C., Hill J.A.S.S. (2011). A theoretical study of hydrogen- and lithium-bonded complexes of F–H/Li and Cl–H/Li with NF_3_, NH_3_, and NH_2_(CH_3_). J. Chem. Phys..

[56] Lipkowski P., Grabowski S.J. (2014). Could the lithium bond be classified as the σ-hole bond—QTAIM and NBO analysis. Chem. Phys. Lett..

[57] Jeffrey G.A. (1997). An Introduction to Hydrogen Bonding.

[58] Scheiner S. (1997). Hydrogen Bonding: A Theoretical Perspective.

[59] Bundhun A., Ramasami P., Murray J.S., Politzer P. (2013). Trends in σ-hole Strengths and Interactions of F_3_MX Molecules (M = C, Si, Ge and X = F, Cl, Br, I). J. Mol. Model..

[60] Bauzá A., Mooibroek T.J., Frontera A. (2013). Tetrel-Bonding Interaction: Rediscovered Supramolecular Force?. Angew. Chem. Int. Ed..

[61] Grabowski S.J. (2014). Tetrel bond σ-hole bond as a preliminary stage of the S_N_2 reaction. Phys. Chem. Chem. Phys..

[62] Zierkiewicz W., Michalczyk M., Scheiner S. (2018). Comparison between Tetrel Bonded Complexes Stabilized by σ and π Hole Interactions. Molecules.

[63] Sundberg M.R., Uggla R., Viñas C., Teixidor F., Paavola S., Kivekäs R. (2007). Nature of intramolecular interactions in hypercoordinate C-substituted 1, 2-dicarba-closo-dodecaboranes with short P···P distances. Inorg. Chem. Commun..

[64] Bauer S., Tschirschwitz S., Lönnecke P., Franck R., Kirchner B., Clark M.L., Hey-Hawkins E. (2009). Enantiomerically pure bis (phosphanyl) carbaborane (12) compounds. Eur. J. Inorg. Chem..

[65] Del Bene J.E., Alkorta I., Sanchez-Sanz G., Elguero J. (2011). Structures, Energies, Bonding, and NMR Properties of Pnicogen Complexes H_2_XP:NXH_2_ (X = H, CH_3_, NH_2_, OH, F, Cl). J. Phys. Chem. A.

[66] Scheiner S. (2011). Can two trivalent N atoms engage in a direct N···N noncovalent interaction?. Chem. Phys. Lett..

[67] Scheiner S., Wysokiński R., Michalczyk M., Zierkiewicz W. (2020). Pnicogen Bonds Pairing Anionic Lewis Acid With Neutral and Anionic Bases. J. Phys. Chem. A.

[68] Bleiholder C., Gleiter R., Werz D.B., Köppel H. (2007). Theoretical Investigations on Heteronuclear Chalcogen—Chalcogen Interactions: On the Nature of Weak Bonds between Chalcogen Centers. Inorg. Chem..

[69] Bleiholder C., Werz D.B., Köppel H., Gleiter R. (2006). Theoretical investigations on chalcogen− chalcogen interactions: What makes these nonbonded interactions bonding?. J. Am. Chem. Soc..

[70] Sanz P., Yañez M., Mó O. (2002). Competition between X···H···Y Intramolecular Hydrogen Bonds and X····Y (X = O, S, and Y = Se, Te) Chalcogen−Chalcogen Interactions. J. Phys. Chem. A.

[71] Wang W., Ji B., Zhang Y. (2009). Chalcogen bond: A sister noncovalent bond to halogen bond. J. Phys. Chem. A.

[72] Alikhani E., Fuster F., Madebene B., Grabowski S.J. (2014). Topological reaction sites–very strong chalcogen bonds. Phys. Chem. Chem. Phys..

[73] Scilabra P., Terraneo G., Resnati G. (2019). The chalcogen bond in crystalline solids: A world parallel to halogen bond. Acc. Chem. Res..

[74] Metrangolo P., Resnati G. (2001). Halogen bonding: A paradigm in supramolecular *chemistry*. Chem. Eur. J..

[75] Cavallo G., Metrangolo P., Milani R., Pilati T., Priimagi A., Resnati G., Terraneo G. (2016). The Halogen Bond. Chem. Rev..

[76] Grabowski S.J. (2014). Boron and other triel lewis acid centers: From hypovalency to hypervalency. ChemPhysChem.

[77] Grabowski S.J. (2015). π-hole bonds: Boron and aluminium lewis acid centers. ChemPhysChem.

[78] Politzer P., Murray J.S. (2013). Halogen Bonding: An Interim Discussion. ChemPhysChem.

[79] Murray J.S., Lane P., Politzer P. (2009). Expansion of the σ-hole concept. J. Mol. Model..

[80] Dong W., Li Q., Scheiner S. (2018). Comparative Strengths of Tetrel, Pnicogen, Chalcogen, and Halogen Bonds and Contributing Factors. Molecules.

[81] Hou M., Yang S., Li Q., Cheng J., Li H., Liu S. (2019). Tetrel Bond between 6-OTX_3_-Fulvene and NH_3_: Substituents and Aromaticity. Molecules.

[82] Könczöl L., Turczel G., Szpisjak T., Szieberth D. (2014). The stability of η^2^-H_2_ borane complexes—A theoretical investigation. Dalton Trans..

[83] Hiberty P.C., Ohanessian G. (1982). Comparison of minimal and extended basis sets in terms of resonant formulas. Application to 1,3 dipoles. J. Am. Chem. Soc..

[84] Dickenson G.D., Niu M.L., Salumbides E.J., Komasa J., Eikema K.S.E., Pachucki K., Ubachs W. (2013). Fundamental Vibration of Molecular Hydrogen. Phys. Rev. Lett..

[85] Crabtree R.H., Siegbahn P.E.M., Eisenstein O., Rheingold A.L., Koetzle T.F.A. (1996). A New Intermolecular Interaction: Unconventional Hydrogen Bonds with Element-Hydride Bonds as Proton Acceptor. Acc. Chem. Res..

[86] Belkova N.V., Shubina E.S., Gutsul E.I., Epstein L.M., Eremenko I.L., Nefedov S.E. (2000). Structural and energetic aspects of hydrogen bonding and proton transfer to ReH_2_(CO)(NO)(PR_3_)_2_ and ReHCl(CO)(NO)(PMe_3_)_2_ by IR and X-ray studies. J. Organomet. Chem..

[87] Custelcean R., Jackson J.E. (1998). Topochemical Control of Covalent Bond Formation by Dihydrogen Bonding. J. Am. Chem. Soc..

[88] Grabowski S.J. (2001). A new measure of hydrogen bonding strength–ab initio and atoms in molecules studies. Chem. Phys. Lett..

[89] Bader R.F.W. (1990). Atoms in Molecules, A Quantum Theory.

[90] Matta C., Boyd R.J. (2007). Quantum Theory of Atoms in Molecules: Recent Progress in Theory and Application.

[91] Carrol M.T., Chang C., Bader R.F.W. (1988). Prediction of the structures of hydrogen-bonded complexes using the Laplacian of the charge density. Mol. Phys..

[92] Carrol M.T., Bader R.F.W. (1988). An analysis of the hydrogen bond in BASE-HF complexes using the theory of atoms in molecules. Mol. Phys..

[93] Parthasarathi R., Subramanian V., Sathyamurthy N. (2006). Hydrogen bonding without borders: An atoms-in-molecules perspective. J. Phys. Chem. A.

[94] Desiraju G.R., Novoa J.J. (2018). Intermolecular Interactions in Crystals: Fundamentals of Crystal Engineering.

[95] Ziegler T., Rauk A. (1979). CO, CS, N_2_, PF_3_, and CNCH_3_ as σ Donors and π Acceptors. A Theoretical Study by the Hartree-Fock-Slater Transition-State Method. Inorg. Chem..

[96] Velde G.T.E., Bickelhaupt F.M., Baerends E.J., Guerra C.F., van Gisbergen S.J.A., Snijders J.G., Ziegler T. (2001). Chemistry with ADF. J. Comput. Chem..

[97] Frisch M.J., Trucks G.W., Schlegel H.B., Scuseria G.E., Robb M.A., Cheeseman J.R., Scalmani G., Barone V., Mennucci B., Petersson G.A. (2016). Gaussian 16, Revision A.03.

[98] Møller C., Plesset M.S. (1934). Note on an Approximation Treatment for Many-Electron Systems. Phys. Rev..

[99] Woon D.E., Dunning T.H. (1993). Gaussian Basis Sets for Use in Correlated Molecular Calculations. III. The second row atoms, Al-Ar. J. Chem. Phys..

[100] Van Duijneveldt F.B., Van Duijneveldt-van de Rijdt J.G.C.M., Van Lenthe J.H. (1994). State of the Art in Counterpoise Theory. Chem. Rev..

[101] Breneman C.M., Wiberg K.B. (1990). Determining atom-centered monopoles from molecular electrostatic potentials.The need for high sampling density in formamide conformational analysis. J. Comp. Chem..

[102] Szefczyk B., Sokalski W.A., Leszczynski J. (2002). Optimal methods for calculation of the amount of intermolecular electron transfer. J. Chem. Phys..

[103] Todd A., Keith T.K. (2011). AIMAll (Version 11.08.23).

[104] Becke A.D. (1988). Density-functional exchange-energy approximation with correct asymptotic behavior. Phys. Rev. A.

[105] Perdew J.P. (1986). Density-functional approximation for the correlation energy of the inhomogeneous electron gas. Phys. Rev. B.

[106] Grimme S., Antony J., Ehrlich S., Krieg H. (2010). A consistent and accurate ab initio parametrization of density functional dispersion correction (DFT-D) for the 94 elements H-Pu. J. Chem. Phys..

[107] Van Lenthe E., Baerends E.J. (2003). Optimized Slater-type basis sets for the elements 1-118. J. Comput. Chem..

[108] ADF2013, SCM Theoretical Chemistry.

